# Private finance and the threat to global health

**DOI:** 10.1136/bmjgh-2025-019726

**Published:** 2025-06-15

**Authors:** Benjamin M Hunter, David McCoy, Ana Carolina Cordilha, Anna Marriott, Victor Roy, Felix Stein, Benjamin Wood

**Affiliations:** 1 School of Social & Political Sciences, University of Glasgow, Glasgow, UK; 2 United Nations University International Institute for Global Health, Kuala Lumpur, Malaysia; 3 University of Rennes 2, Rennes, France; 4 Oxfam GB, Oxford, UK; 5 University of Pennsylvania, Philadelphia, Pennsylvania, USA; 6 University of Amsterdam, Amsterdam, Netherlands; 7 Deakin University, Melbourne, Victoria, Australia

**Keywords:** Global Health, Health policy, Health systems, Universal Health Care

**Summary**

- The expansion of private finance in global health is pronounced.
- There are fundamental contradictions at the heart of this change, between the health concerns of the poorest communities and the financial returns of wealthy investors.
- The global health community needs to do more to learn about how private finance works and to challenge common fallacies and false narratives.
- Advocacy must be strengthened for existing alternative models of financing and governance, including debt justice and tax justice initiatives.

Investment is a defining word of the moment. Last year’s inaugural ‘investment round’ for WHO^1^ followed calls from its Director-General to see ‘health not as a cost, but an investment’.^2^ The Lancet Commission on Investing in Health was met with acclaim at its offering up of health as a ‘compelling investment opportunity’.^3^ The recent collapse of funding for global health causes and institutions has lent added urgency to the search for new sources of investment. However laudable the intentions of maximising money for global health and its key institutions may be, the approach adopted has so far largely skirted key questions such as *from whom*, *on what terms* and *who ultimately benefits?* It leaves countries and institutions vulnerable to commercial interests and extractive practices that run counter to the mission of global health. This is the subject of our new report: *Private Financial Actors and Financialisation in Global Health*.^4^

The expansion of private finance in global health is pronounced and relatively new.^5 6^ While there has always been attention paid to private *financing*, which includes out-of-pocket payments and private health insurance, a recent emphasis on private *finance* has seen the global health community welcoming ‘investment’ from private equity firms, hedge funds and investment banks. This is an investment that expects a financial return for its investors that exceeds or, at the very least matches, other investment opportunities. Among international organisations and bilateral agencies, there has been interest in using innovative financial mechanisms to draw private finance into global health. This includes Gavi’s International Finance Facility for Immunisation (IFFIm) and the World Bank Group’s Global Financing Facility and Pandemic Emergency Financing Facility (PEF). National policy actors too have looked to partnerships with private financial actors to pay for new infrastructure and continue service provision. Private financial actors, for their part, have shown keen interest in opportunities for profit in an era of escalating health needs, with large investments being made in companies involved in pharmaceuticals, health technologies, healthcare provision and insurance.

In promoting private finance, international organisations and bilateral agencies have glossed over the fundamental contradictions of promoting health for the poorest communities while enabling double-digit profits by wealthy investors. The pivot to private finance creates opportunities for investors *to take money out of global health* through mechanisms such as interest payments, share buybacks, dividends, subsidies and equity trading. A study on IFFIm identified USD 879 million in interest payments made to bondholders over a 13 year period from government aid budgets^7^; the windfall profits achieved by vaccine manufacturers Moderna, Pfizer and BioNTech during the COVID-19 pandemic resulted in dividends and buybacks totalling USD 13 billion.^8^ Fund managers, investment banks, insurers, auditors and law firms are used to facilitate transactions, resulting in further extraction of money. For example, fund manager Abraaj paid itself USD 40 million during the first 18 months of a Growth Health Markets Fund that government and multilateral backers claimed would build affordable high-quality health systems in South Asia and Sub-Saharan Africa.^9^ These processes of extraction not only enrich the world’s wealthiest people, with the richest 1% of people owning 43% of global financial assets,^10^ but also strengthen a financial system where speculation causes harm to social equality, health and the environment.^11^

Moreover, to pave the way for private finance, governments and public agencies are reorienting their policy approaches to prioritise investors’ needs, even at the detriment of health objectives. In the case of PEF, overly restrictive criteria were applied to delay the release of investors’ money to countries experiencing emergency disease outbreaks; PEF only paid out 4 months after the first cases of COVID-19 were reported. The COVID-19 vaccine facility (COVAX) prioritised concern with corporate financial risk (for pharmaceutical companies) over public health risks to participating countries.^12^ Infrastructure public–private partnerships have proved a fiscal disaster for governments locked into multi-decadal contracts with escalating payment schedules: payments by Lesotho’s government to a private consortium ended up consuming half the country’s health budget^13^; in Turkiye, payments for ten hospitals exceeded 25% of the health budget.^14^ Pharmaceutical and biotechnology companies maximise company value and shareholder wealth through strategic acquisitions and navigating intellectual property regimes. Corporate healthcare providers follow a strategy of growth through acquisition that drives market consolidation and converts non-profit hospitals into for-profit models that engage in aggressive and unsafe cost-cutting and the provision of unnecessary tests and treatments.^15^ Public financing for global health is being re-framed to ‘de-risk’ private investment, often via costly ‘blended finance’ mechanisms.^12^

A press report, out in January,^16^ shone light on the human costs of ‘investment’. It documents rights abuses taking place in corporate hospitals in Uganda and The Philippines, following similar investigations by Oxfam in India and Kenya.^17^ They reveal how the need to generate revenue to appease investors translates into practices such as denying emergency medical care and detaining users for non-payment of bills. The corporate sector caters to wealthier groups and excludes the majority, even though the hospitals featured in the reports have benefited from public investments from the World Bank Group and UK, French and German government-owned development institutions.

This is all happening in the name of 'investment’, a seemingly benign catch-all term that obscures important details of who pays for and who benefits from the profits of private investment. We need to discuss the scale at which private finance is entering the health sector and the seriousness of the dangers associated with its expansion. While there are health financing gaps that must be urgently filled, this must not be a Trojan horse by which neo-colonial, exploitative and extractive forms of health privatisation are enabled, and which frequently enriches financial hubs in the Global North. The need to learn about how private finance works and to constantly challenge common fallacies and false narratives used to promote private finance in health is why we included a glossary of terms and concepts in our report.

This is not to deny the existence of a funding crisis facing many countries and institutions in global health. But there are other more just and equitable ways to address this. This includes improvements achieved through healthcare financing reforms and health systems strengthening efforts; reducing the large transaction costs involved in the crowded and fragmented global health industrial complex; reducing the excessive market prices of essential medicines, vaccines and other technologies; and rebuilding systems for international cooperation. More broadly, public revenue for health can and must be expanded through measures such as the cancellation of unsustainable debt, progressive use of special drawing rights and tackling both legal and illegal tax practices that undermine public revenue.

So far, private finance has been promoted in an uncritical manner and in global health contexts where there are insufficient safeguards for preventing large-scale profiteering, exploitation of workers and service users and a weakening of already fragile healthcare systems. The latter includes the often smaller, community-based and more accountable private providers on whom many people rely and who are also vulnerable to the impacts of corporatisation and capture by private finance. Proposals to use private finance solutions in future need to be accompanied by much stronger evidence of alignment with health equity concerns and with tighter regulation and public accountability mechanisms. There can be a productive role for private finance in society, but safeguards are needed to prevent harm, especially when public funds or public risk-sharing strategies are involved.

## Data Availability

No data are available.
